# Adsorption Characteristics of Hair Dyes Removal from Aqueous Solution onto Oak Cupules Powder Coated with ZnO

**DOI:** 10.3390/ijms231911959

**Published:** 2022-10-08

**Authors:** Alaa M. Al-Ma’abreh, Razan Ataallah Abuassaf, Dareen A. Hmedat, Manal Alkhabbas, Gada Edris, Samer Hasan Hussein-Al-Ali, Samer Alawaideh

**Affiliations:** Department of Chemistry, Faculty of Science, Isra University, P.O. Box 22, Amman 11622, Jordan

**Keywords:** adsorption, hair dyes, ZnO, oak, kinetics, thermodynamic

## Abstract

Three hair dyes of Arianor madder red 306003 (R), Arian or Straw Yellow 306005 (Y), and Arianor ebony 306020 (E) were removed from an aqueous solution in a batch mode using a powder of oak cupules coated with ZnO (COZ). The COZ-adsorbent material was characterized in terms of XRD, FT-IR, and SEM analysis. The best conditions for the uptake of hair dyes by COZ were investigated. For Y dye, the best uptake was estimated on 0.06 g of COZ at 7.0 pH for 150 min. The E dye uptake requires 120 min on 0.05 g of COZ at 9.0 pH. For E hair dye, kinetic data revealed a pseudo-first-order model for E hair dye and a pseudo-second-order model for R and Y. Equilibrium data exhibited consistency with the Langmuir isotherm model for the adsorption of E dye onto COZ, and the Freundlich isotherm model for the adsorption of R and Y hair dyes onto COZ. Isotherms models of D-R and Temkin were also examined. The thermodynamic parameters (−ve ∆G and +ve ∆H and ∆S) demonstrated that the removal of hair dyes by COZ is spontaneous, endothermic, and feasible. The adsorption capacity of COZ for R, Y, and E uptake was found to be 55.5, 52.6, and 135.1 mg·g^−1^, respectively. Furthermore, COZ reusability was demonstrated after five cycles of regeneration, with a negligible decline in adsorption extent (13.08%, 13.85, and 10.20% for R, Y, and E, respectively) in comparison to its initial capacity.

## 1. Introduction

Monitoring pollution is now one of society’s top priorities. The environment is listed as being threatened by organic dyes. Organic dyes are introduced to aquatic systems by the effluents of several sectors, including paint, textile, medicinal, and biotechnology. These colors do not naturally degrade and persist in aquatic systems [1]. The production of aromatic molecular dyes from hydrocarbons such as benzene, toluene, etc. raises unique environmental challenges [2]. Per the characteristics they give water solutions, dyes are categorized as cationic, anionic, or nonionic. The inclusion of amine groups in the structure makes azo dyes particularly poisonous. Before releasing these azo dyes into water effluents, their levels should be as low as feasible because they may cause harm to both the environment and human health [1]. Chemical precipitation, membrane filtration, ion exchange, electrolysis, coagulation, solvent extraction, reverse osmosis, and electrocoagulation are examples of methods and processes utilized to remove contaminants [3]. It was reported that the adsorption methods are more efficacious than other physical and chemical remedies [4]. Among various purifying methods, adsorption is versatile, ubiquitous, inexpensive, affordable, safe for human health, ecologically friendly, and effective. For removing dyes from an aqueous solution, numerous studies relying on the adsorption approach employing natural surfaces were conducted. Zinc oxide nanoparticles are promising adsorption materials for removing hazardous contaminants such as dyes. The reasons are related to ZnO nanoparticles’ appealing properties such as low cost, non-toxicity, chemical stability, thermal stability, photo-stability, and high UV absorption [5]. The high isoelectric point of ZnO (9.5) improves its surface positive (+ve) charge. ZnO nanoparticles have recently been used to coat various adsorbents, such as zeolite [6]. Under convenient conditions, ZnO nanoparticles are highly effective at removing anionic azo-dyes such as amaranth and methyl orange from aqueous systems [7]. The use of an assisted microwave technique to Coat chitosan with ZnO nanoparticles results in a significant improvement in the removal of methylene blue (MB) dye from an aqueous solution [8]. Under optimal conditions, nanostructured ZnO material demonstrated remarkable efficiency in the removal of cationic and anionic dyes such as malachite green, acid Fuchsin, and congo red [9].

**Scheme 1:**
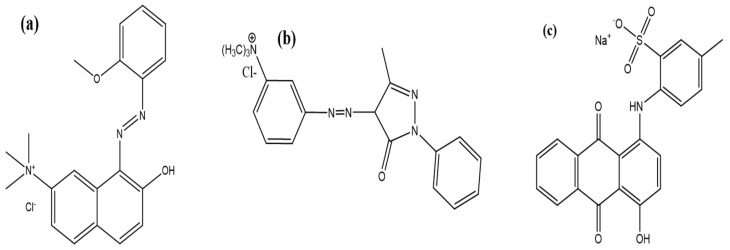
The chemical structure of (**a**) Arianor madder red 306003 (**R**), (**b**) Arianor Straw Yellow 306005 (**Y**), and (**c**) Arianor ebony 306020 (E).

In this study, an affordable adsorbent material for the uptake of hair dyes from an aqueous solution was prepared from a powder of oak cupules coated with ZnO. The effectiveness of the adsorption process was optimized by analyzing the parameters of adsorbent dosage, initial concentration, contact time, temperature, and pH. Kinetic, isothermal, and thermodynamic studies were carried out to deduce the equilibrium and mechanism of the adsorption process. Scheme 1 shows the chemical structure of three colors of hair dyes, Arianor madder red 306003 (R), Arianor Straw Yellow 306005 (Y), and Arianor ebony 306020 (E). 

## 2. Results and Discussion

### 2.1. Characterization of Adsorbent Material

Techniques of FT-IR, SEM, and XRD were employed to characterize the adsorption process of the three hair dyes (R, Y, and E) onto COZ. 

**Figure 1:**
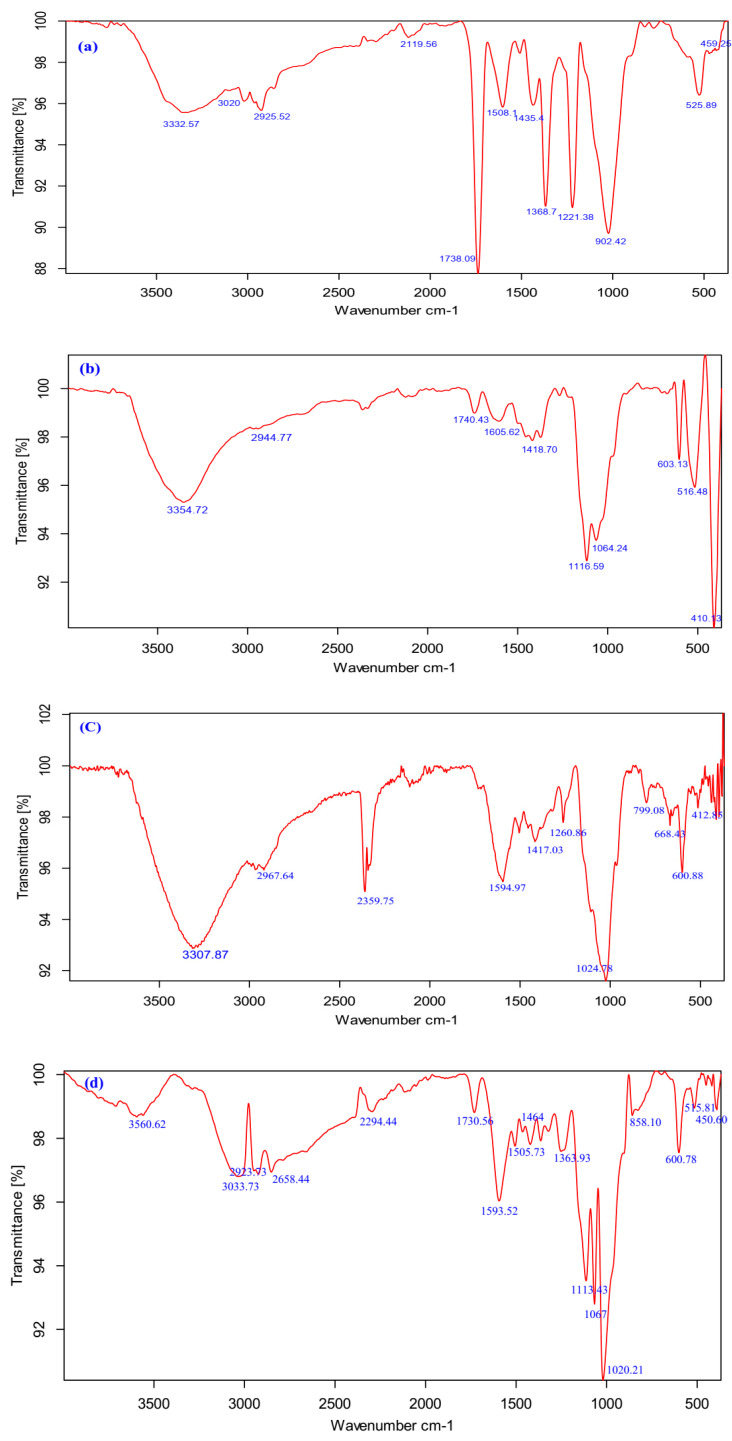

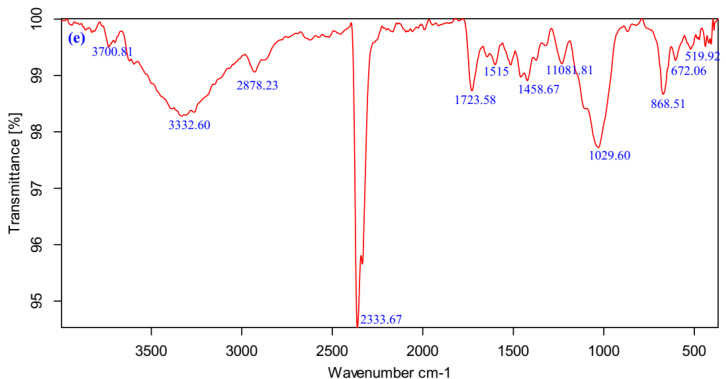
FT-IR analysis of (**a**) raw oak cupules powder and (**b**) coated oak cupules powder with ZnO [COZ]; (**c**) red hair dye onto COZ [COZR], (**d**) Yellow hair dye onto COZ [COZY], and (**e**) Ebony dye onto COZ [COZE].

The FT-IR analysis was utilized to recognize and categorize functional groups that serve as active sites on the surface of COZ. The FT-IR spectra of natural oak cupules powder Figure 1a shows many bands at 3332.57, 3020, 2925.52, 2119.56, 1738.09, 1603.04, 1508.1, 1435.4, 1368.7, 1221.38, 902.42, 525.89, and 459.25 cm^−1^. The functional groups assigned to these bands are detailed in Table 1 [10,11]. When comparing Figure 1b to the spectrum of oak cupules powder (Figure 1a), there are significant shifts and changes in intensity for all bands, particularly those at 525.89 cm^−^^1^, which shifts to 516.48 and at 459.69 shifts to 410.13. These changes in intensity and wavenumbers can be attributed to the interactions between oak cupules and the ZnO. According to Figure 1c–e, loaded hair dyes on COZ displayed fluctuation in intensity when compared to COZ, with a little shift in the majority of the bands. 

**Figure 2:**
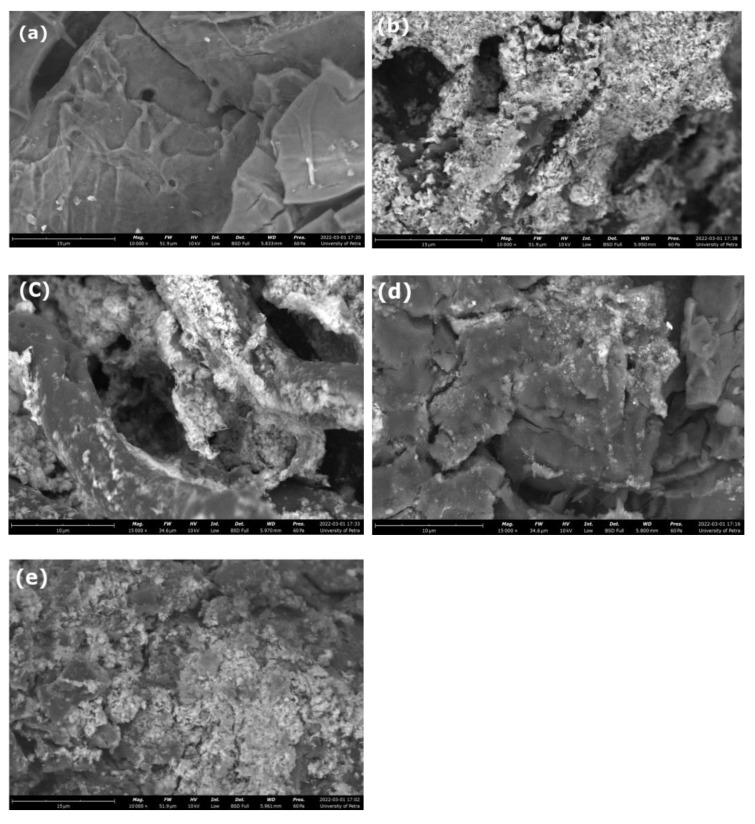
SEM analysis of (**a**) oak cupule powder **O**, (**b**) oak cupule powder coated with ZnO **COZ**, (**c**) **COZR**, (**d**) **COZY**, (**e**) **COZE**.

Table 1 FT-IR results for all samples of **O**, **COZ**, **COZR**, **COZY**, and **COZE**:

The surface morphology of **O, ZO, COZ, COZR, COZY**, and **COZE** is portrayed by SEM. The adsorption process of the three hair dyes **R**, **Y**, and **E** onto **COZ** are depicted in SEM images (Figure 2a–e), in which the accumulation of dye moieties on the COZ surface is clearly evident.

**Figure 3:**
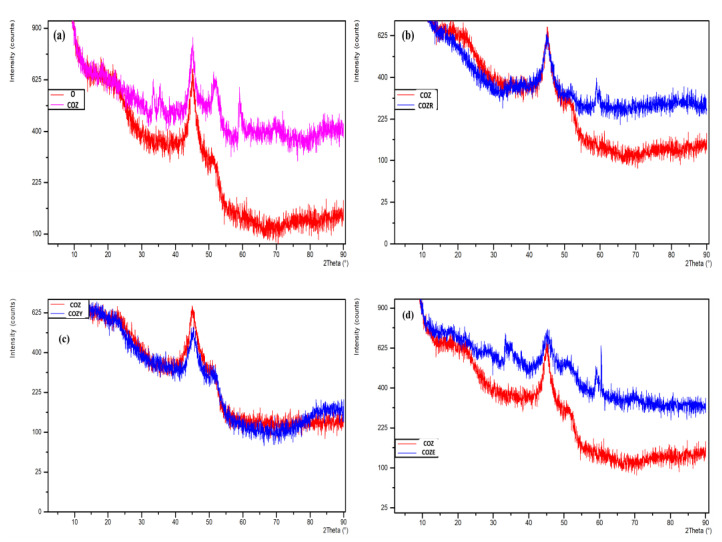
XRD analysis of (**a**) oak [**O**] before coated and after coated with ZnO [**COZ**], (**b**) **COZ** before and after adsorption with red hair dye [**COZR**], (**c**) **COZ** before and after adsorption with yellow hair dye [**COZY**], and (**d**) **COZ** before and after adsorption with Ebony hair dye [**COZE**].

The adsorption of three hair dyes (R, Y, and E) onto COZ was discerned using XRD. Figure 3 depicts the obtained spectra. The difference in intensity and broadening between spectra before and after adsorption is striking. 

### 2.2. Batch Adsorption 

#### 2.2.1. Adsorbent Dosage

The adsorption process is impacted by the variation in adsorbent dosage. The adsorption capacity was investigated using the following equation [9]:

(1) $$q_{e}=\frac{\left(C_{i}-C_{eq}\right)V}{m}\;\;$$

where *q_e_* (mg·g^−1^) is the equilibrium adsorbed amount, *C_i_* and *C_eq_* (mg·L^−1^) are the initial and equilibrium concentrations, respectively. *V* (L) is the volume of solution.

**Figure 4:**
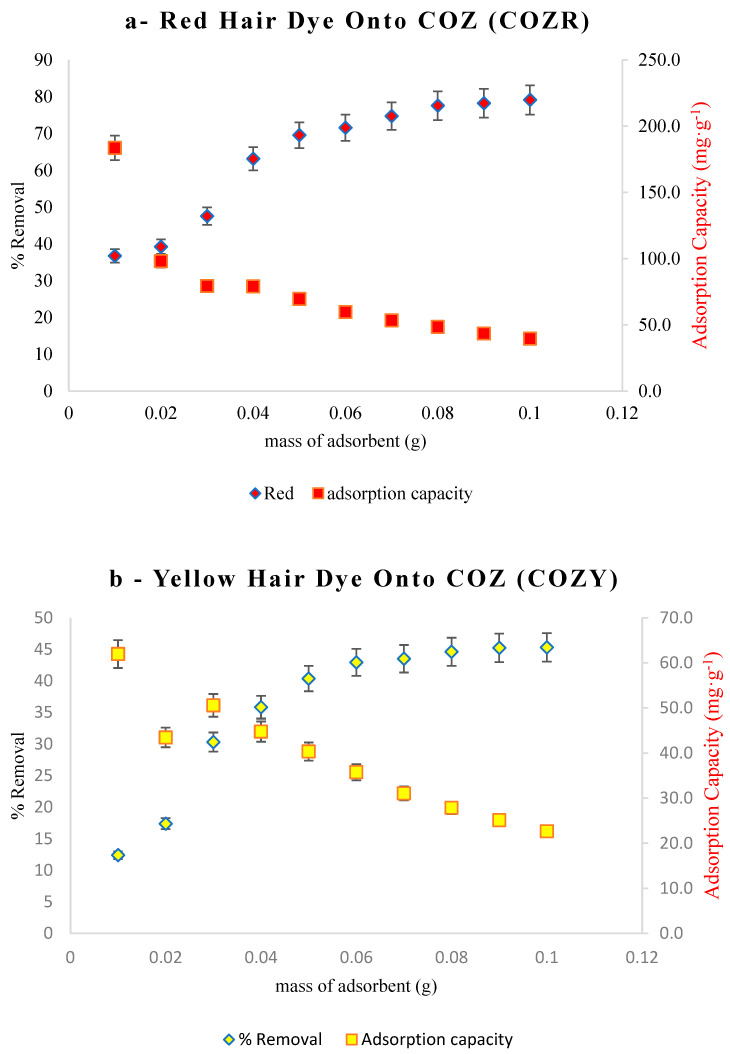

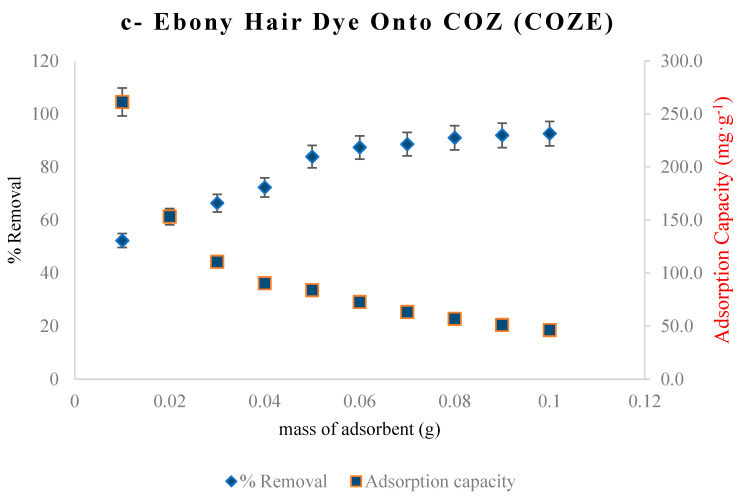
Effect of adsorbent dosage on the adsorption of (**a**) red hair dye onto COZ (**b**) yellow hair dye onto COZ, and (**c**) ebony hair dye onto COZ [adsorbent dosage = 0.01–0.1 g, C_i_ = 50 mg·L^−1^; pH = 7.0, and contact time = 60 min, and T = 25 ± 1 °C, speed = 150 rpm].

The adsorbent dosage used in the experiment ranged from 0.01 to 0.1g. The optimal conditions of the experiment were fixed at 150 rpm shaking for 60 min, at a concentration of 50 mg·L^−1^, a temperature of 25 ± 1 °C, and a pH of 7.0. The results are emphasized in Figure 4a–c. The R, Y, and E all experienced an increase in hair dye uptake to 0.07 g, 0.06 g, and 0.05 g, respectively. As the dosage of COZ increases, the equilibrium adsorption capacity begins to decline. This behavior could be explained by the prevalence of numerous functioning sites on the COZ surface.

#### 2.2.2. Contact Time

**Figure 5:**
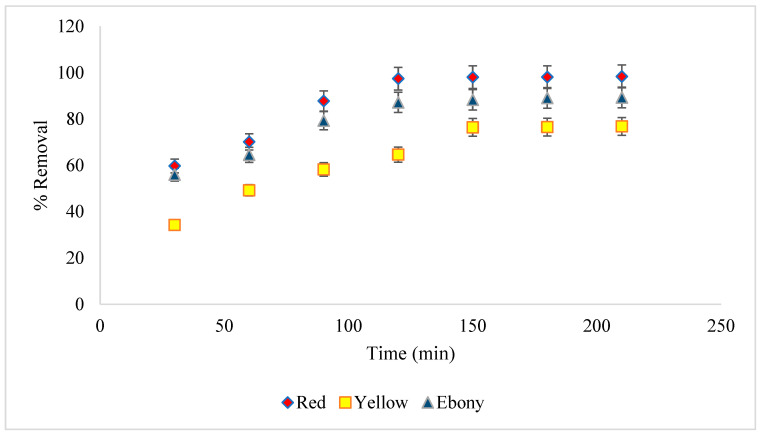
Effect of contact time on the adsorption of hair dyes [dosage = 0.07 g R, 0.06 g Y, & 0.05 E, time = 20–210 min, C_i_ = 50 mg·L^−1^; pH = 7.0, and T = 25 ± 1 °C, speed = 150 rpm].

The performance of COZ adsorbent in removing R, Y, and E dyes was investigated considering the impact of contact time. The results of the experiments under fixed conditions were emphasized in Figure 5. The percentage removal of hair dyes will steadily increase until an equilibrium is achieved. In the earliest stages, the removal percentages sizably increased due to the abundant and sizeable accessible functioning sites on the COZ surface. Results demonstrated that R and E dyes required 120 min to equilibrate, whereas Y dye takes 150 min.

#### 2.2.3. Adsorbate Concentration

**Figure 6:**
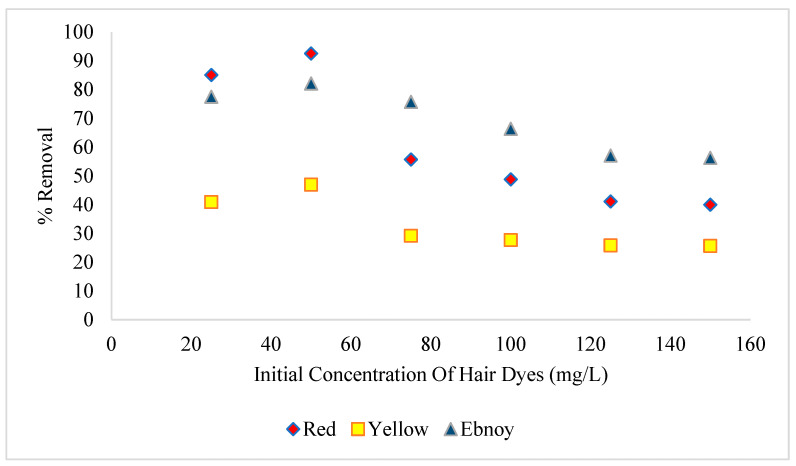
Effect of adsorbate initial concentration (25–150 mg·L^−1^) on the adsorption of red, yellow, and ebony hair dyes; [dosage = 0.07 g R, 0.06 g Y, & 0.05 E, pH = 7.0, contact time = 120 min for R and E, 150 min for Y, T = 25 ± 1 °C, speed = 150 rpm].

The impact of initial dye concentration variance (25–150 mg·L^−1^) on COZ performance in removing R, Y, and E dyes was investigated. The results are emphasized in Figure 6. In the earliest stages, the results revealed a considerable rise. The COZ adsorbent showed its best performance at a concentration of 50 mg·L^−1^ for each of the three dyes (R, Y, and E). The saturation of functioning sites on the COZ adsorbent increased substantially, resulting in a slight decline in the adsorbent capacity [12,13]. 

#### 2.2.4. pH

**Figure 7:**
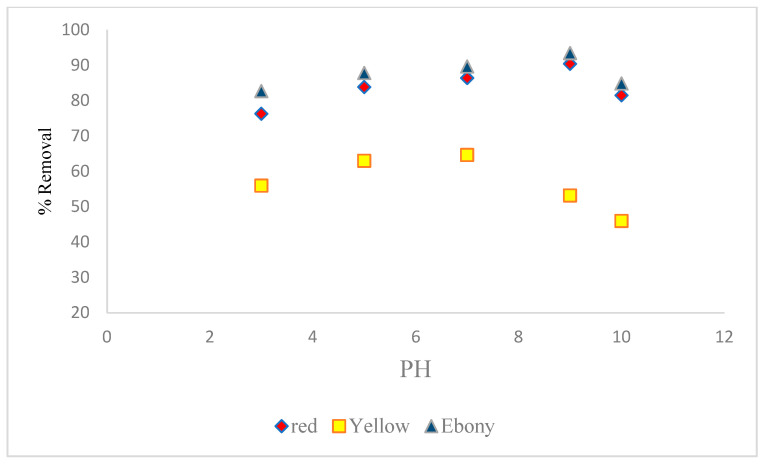
Effect of pH on the adsorption of hair dyes (red, yellow, and ebony) onto COZ; [dosage = 0.07 g R, 0.06 g Y, & 0.05 E, C_i_ = 50 mg·L^−1^, contact time = 120 mint. For R and E, 150 mint. For Y, T = 25 ± 1 °C, speed = 150 rpm].

The zero-charge point (pH_pzc_) was determined by shaking 50 mL of 0.1 M NaCl solutions with 0.15 g COZ for 24 h. at various pH values (2, 4, 6, 8, 10, and 12). The pH_f_ vs pH_i_ plot indicated a pH_pzc_ value of 9.5. When the pH exceeds the pHpzc, the adsorbent surface charge is negative (−ve), and vice versa [14]. According to Figure 7, the batch adsorption experiments were conducted under optimal conditions and at a pH range between 3 and 10. R and E hair dyes’ adsorption functionality was remarked to modestly rise when the pH value increased from 3.0 to 9.0. For Y dye, the adsorption capacity increases to neutral before declining at basic. At low pH, the rather modest adsorption of hair dyes onto COZ adsorbent is due to the significant amount of H+ that competes with the dye cation in adsorption [15]. Further increases in pH result in more negatively charged surfaces. Because of these negatively charged surfaces, the affinity between the positively charged (+ve) dye molecule and the adsorbent is attenuated [16]. The optimal pH for R and E dye adsorption by COZ was realized to be 9.0, and 7.0 for Y. 

#### 2.2.5. Adsorption Kinetic Studies

Pseudo-first-order and pseudo-second-order kinetics were employed to investigate the adsorption of hair dyes (R, Y, and E) onto COZ. 

The following pseudo-first-order kinetic linear equation was employed [17]:

(2) $$\log\left(q_{e}-q_{t}\right)=\log\;(q_{e})-\left(\frac{k_{1}}{2.303}\right)t$$

where, *k*_1_ expresses the rate constant (min^−^^1^), *q_e_* is the equilibrium adsorbed amount of material per unit mass of adsorbent (mg·g^−^^1^), and *q_t_* is the equilibrium adsorbed amount of material per unit mass of adsorbent at time t (mg·g^−^^1^).

The following pseudo-second-order kinetic linear equation was employed [18]:

(3) $$\frac{t}{q_{t}}=\frac{1}{k_{2}q_{e}^{2}}-\frac{1}{q_{e}}t$$

where, *k*_2_ is the rate constant (g·mg^−^^1^ min^−^^1^), *q_e_* is the equilibrium adsorbed amount of material per unit mass of adsorbent (mg·g^−^^1^), and *q_t_* is the equilibrium adsorbed amount of material adsorbed per unit mass of at time *t* (mg·g^−^^1^). The value of *k*_2_ was obtained using the slope and intercept from the plot of *t*/*q_t_* versus *t*.

**Figure 8:**
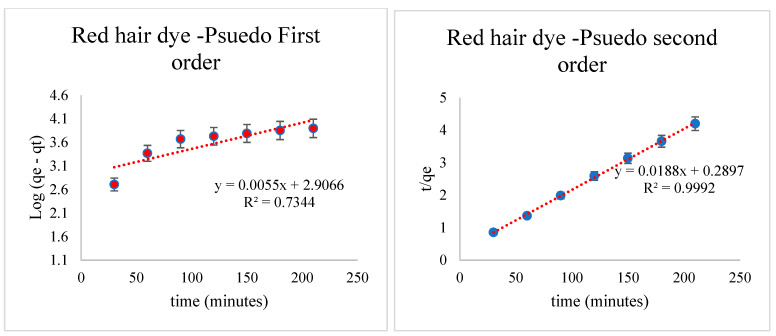
Kinetic studies of the adsorption of red hair dye onto COZ, [dosage = 0.07 g R, 0.06 g Y, & 0.05 E, C_i_ = 50 mg·L^−1^, pH = 7.0, contact time = 120 min for R and E, 150 min for Y, T = 25 ± 1 °C, speed = 150 rpm].

**Figure 9:**
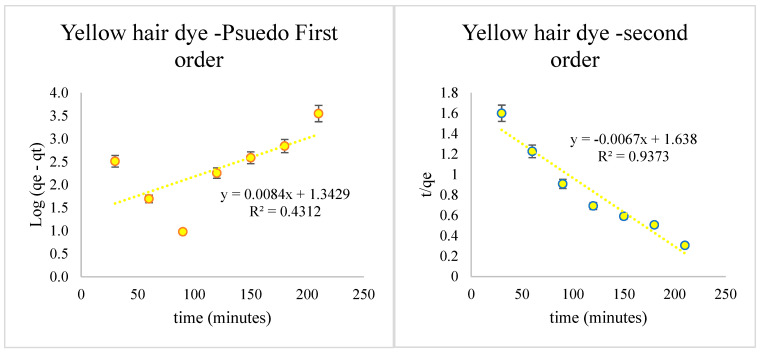
Kinetic studies of the adsorption of yellow hair dye onto COZ, [dosage = 0.07 g R, 0.06 g Y, & 0.05 E, C_i_ = 50 mg·L^−1^, pH = 7.0, contact time = 120 min for R and E, 150 min for Y, T = 25 ± 1 °C, speed = 150 rpm].

**Figure 10:**
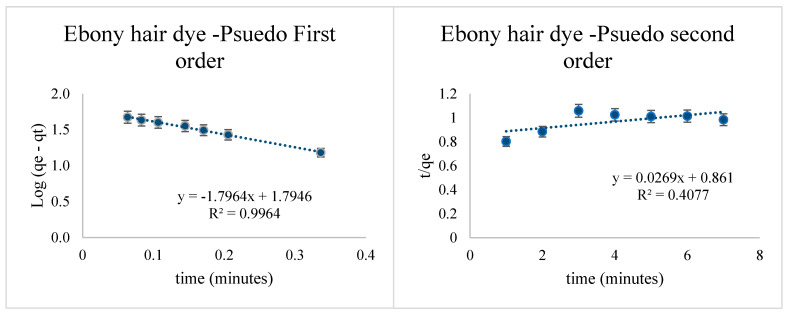
Kinetic studies of the adsorption of ebony hair dye onto COZ, [dosage = 0.07 g R, 0.06 g Y, & 0.05 E, C_i_ = 50 mg·L^−1^, pH = 7.0, contact time = 120 mint. for R and E, 150 mint. for Y, T = 25 ± 1 °C, speed = 150 rpm].

Rate constants for the three hair dye were obtained from the plots in Figure 8, Figure 9 and Figure 10 and then compiled in Table 2. The kinetic model that best fits the adsorption of hair dyes onto COZ was determined based on R^2^ values. R^2^ values were employed to identify the kinetic model that best fits the adsorption of hair dyes onto COZ. Thus, according to Figure 8 and Figure 9, the pseudo-second-order is adequate to describe the adsorption of R and Y hair dyes onto COZ. However, as demonstrated in Figure 10, the pseudo-first-order is adequate to describe the adsorption of E hair dye onto COZ.

The following three sequent steps could be employed to demonstrate the dynamic mechanism of the adsorption process: (i) film diffusion, in which the adsorbate moieties transmit from the bulk solution to the exterior surface of the adsorbent; (ii) particle diffusion, in which adsorbate moieties diffusion into the pore of adsorbent; and (iii) adhering of adsorbate moieties onto the pores interior surfaces [19]. Intra-particle diffusion (Equation (4)) and Boyd (Equations (5) and (6)) models were employed to investigate the rate-limiting step that controlled the adsorption of hair dyes (R, Y, and E) onto COZ [20].

(4) $$q_{t}=K_{id}t^{\frac{1}{2}}+C$$

where *q_t_* is the adsorbed amount of the hair dye in mg·g^−1^, *k_id_* is the rate constant in mg/g min^1/2^, and *t*^1/2^ is the square root of time in min^1/2^.

(5) $$B_{t}=-0.4977-\ln\left(1-F\right)$$

(6) $$F=\frac{q_{t}}{q_{o}}$$

where *B_t_* is a mathematical function of *F* that expresses the fraction of adsorbate moieties that are adsorbed at any time *t*, *q_o_* is the amount of hair dye adsorbed at the infinite time (mg·g^−1^), and q_t_ expresses the amount of hair dye adsorbed at any time *t* (min).

**Figure 11:**
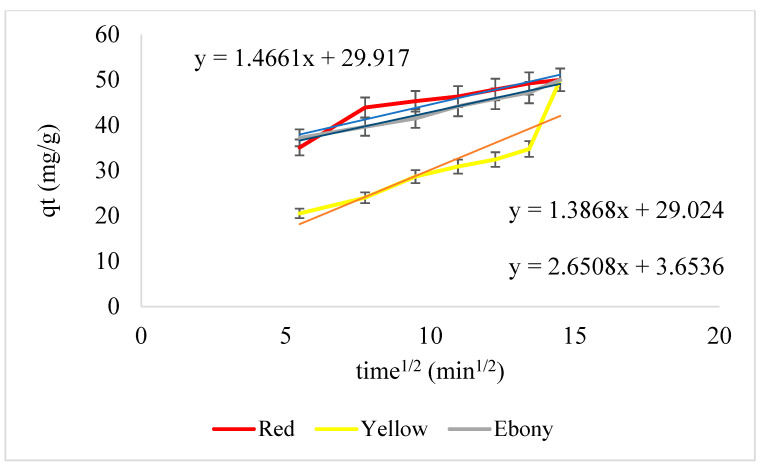
Intra-Particle Diffusion Plot for R, Y, and E hair dyes uptake onto COZ adsorbent.

The nonlinearity emphasized in a plot of *q_t_* versus *t*^1/2^ (Figure 11) indicates adsorption cruised into two steps for the three hair dyes (R, Y, and E). The first straight line for each of the three hair dyes is explained by chemisorption, and the second is controlled by an intra-particle diffusion mechanism.

**Figure 12:**
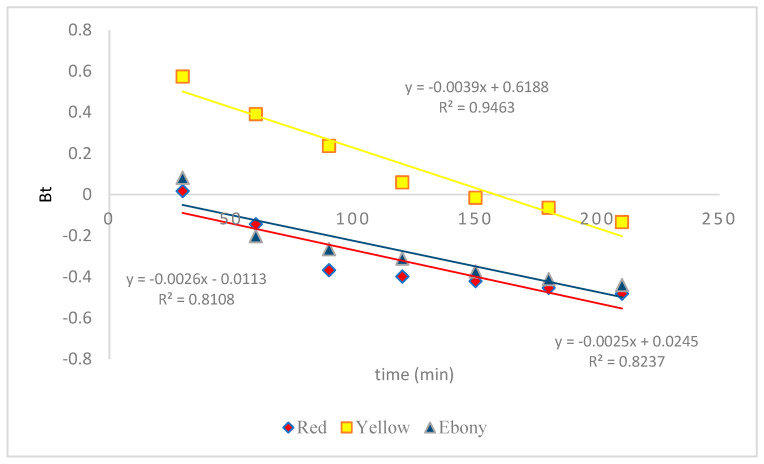
Boyd Plot for R, Y, and E hair dyes uptake onto COZ adsorbent.

The nonlinear plot of Bt versus time in the Boyd model (Figure 12) confirms the intra-particle model results. Boyd’s model implies that external mass transfer mainly governs the rate of the adsorption process.

#### 2.2.6. Adsorption Isotherms

Isotherms of the Langmuir, Freundlich, Dubinin—Radushkevich (D-R), and Temkin models were applied to comprehend the mechanism of the adsorption of hair dyes onto adsorbent material. The Langmuir isotherm model’s linear equation is represented by the following equation [18]:

(7) $$\frac{C_{e}}{q_{e}}=\frac{1}{k_{L}q_{m}}+\frac{C_{e}}{q_{m}}$$

where *q_e_* is the equilibrium adsorbed amount of the material (mg·g^−^^1^), *q_e_* is the equilibrium adsorbed amount of the material (mg·g^−^^1^), *K_L_* is the Langmuir isotherm constant related to the energy of adsorption and used to determine the affinity of the adsorbate to the adsorbent surface, and *C_e_* is the equilibrium concentration of material in the solution (mg·L^−^^1^).

The slope and intercept of the linear plot of *C_e_*/*q_e_* vs. *Ce* were employed to figure out the values of the *K_L_* and *q_m_* constants. The parameter R_L_ was estimated using Equation (8) and thus, could be used to anticipate the adsorbent efficacy. The process is considered to be irreversible if *R_L_* is just zero, favorable if *R_L_* is below one, linear if *R_L_* is just one, and unfavorable if *R_L_* is higher than unity:

(8) $$R_{L}=\frac{1}{1+k_{L}C_{i}}$$

where, *K_L_* is the Langmuir isotherm constant determined in Equation (7), and Ci is the initial concentration of the adsorbate.

The Freundlich isotherm model linear equation is represented by the following:

(9a) $$\log q_{e}=\log K_{f}+\frac{1}{n}\log C_{e}$$

where *q_e_* is the equilibrium concentration of the solid phase material per gram of adsorbent (mg·g^−^^1^), *C_e_* is the equilibrium concentration of the material in the bulk phase (mg·L^−^^1^), *K_f_* is the Freundlich isotherm constant (mg·g^−^^1^), and n is the intensity of adsorption.

The slope and intercept of the linear plot of log *q_e_* vs. *C_e_* were employed to figure out the values of the *K_f_* and *n* constants.

**Figure 13:**
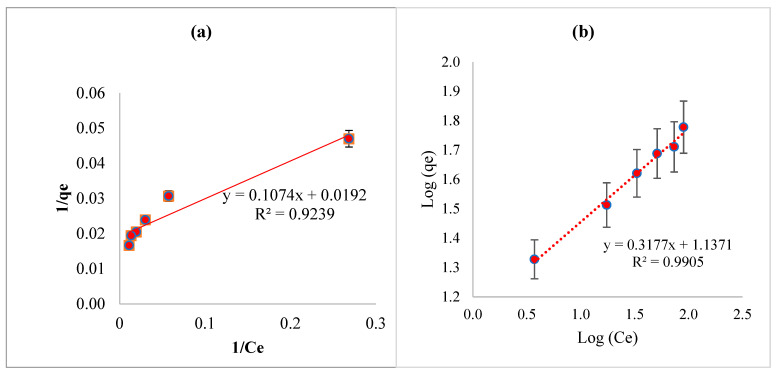
(**a**) Langmuir isotherm of red hair dye onto COZ, and (**b**) Freundlich isotherm of red hair dye onto COZ.

**Figure 14:**
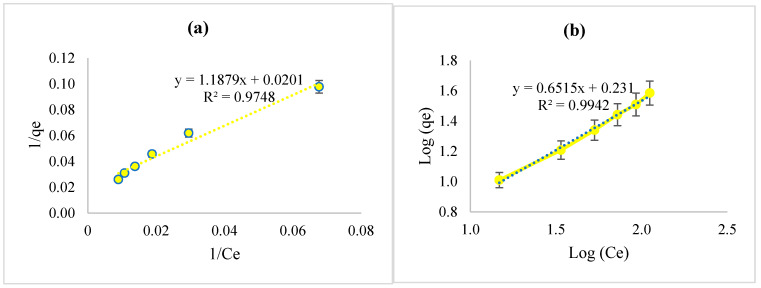
(**a**) Langmuir isotherm of yellow hair dye onto COZ, and (**b**) Freundlich isotherm of yellow hair dye onto COZ.

**Figure 15:**
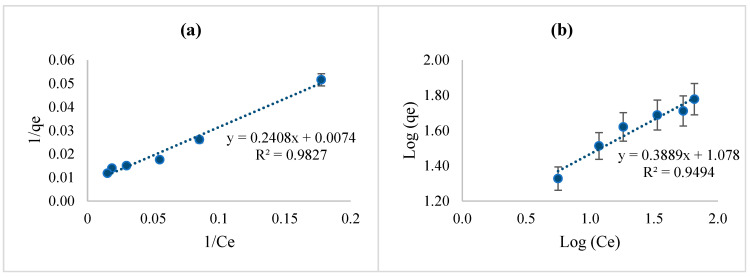
(**a**) Langmuir isotherm of ebony hair dye onto COZ, and (**b**) Freundlich isotherm of ebony hair dye onto COZ.

The Langmuir and Freundlich isotherms for hair dyes onto COZ were emphasized in Figure 13, Figure 14 and Figure 15. All isotherms constants for the adsorption of hair dyes (R, Y, and E) onto COZ were calculated (Table 3). According to the dimensional parameters for the Langmuir isotherm (R_L_), the adsorption of the three hair dyes (R, Y, and E) onto COZ is a favorable process. In accordance with the correlation coefficients (R^2^), the adsorption of R and Y hair dyes onto COZ is adequate to the Freundlich isotherm, as emphasized in Figure 13 and Figure 14**.** On the other hand, the adsorption of E is supposed to follow the Langmuir isotherm, as emphasized in Figure 15. Thus, according to Table 3, the Freundlich constant (n) value is significantly higher than one, implying that R and Y hair dyes preferentially bind to heterogeneous COZ surface [20].

For the Dubinin—Radushkevich (D-R) isotherm model, the equation used was as follows [19]:

(9b) $$\ln q_{e}=\ln Q_{D}-B_{D}\varepsilon^{2}$$

where *Q_D_* is the maximum capacity in mol/g (theoretical), and *B_D_* is a constant of the D-R model in mol^2^/KJ^2^.

*Ɛ* is Polanyi potential and it is expressed by:

(10) $$\varepsilon =RT\ln\left(1+\frac{1}{C_{e}}\right)\;$$

The mean energy of the adsorption process in KJ/mol is calculated by:

(11) $$E=\frac{1}{\sqrt{2B_{D}}}$$

For the Temkin isotherm model, the equation used was as follows [18]:

(12) $$q_{e}=B\ln A+B\ln C_{e}$$

where *A* is the equilibrium binding constant in g^−1^, and *B* is the constant related to the heat of adsorption.

**Figure 16:**
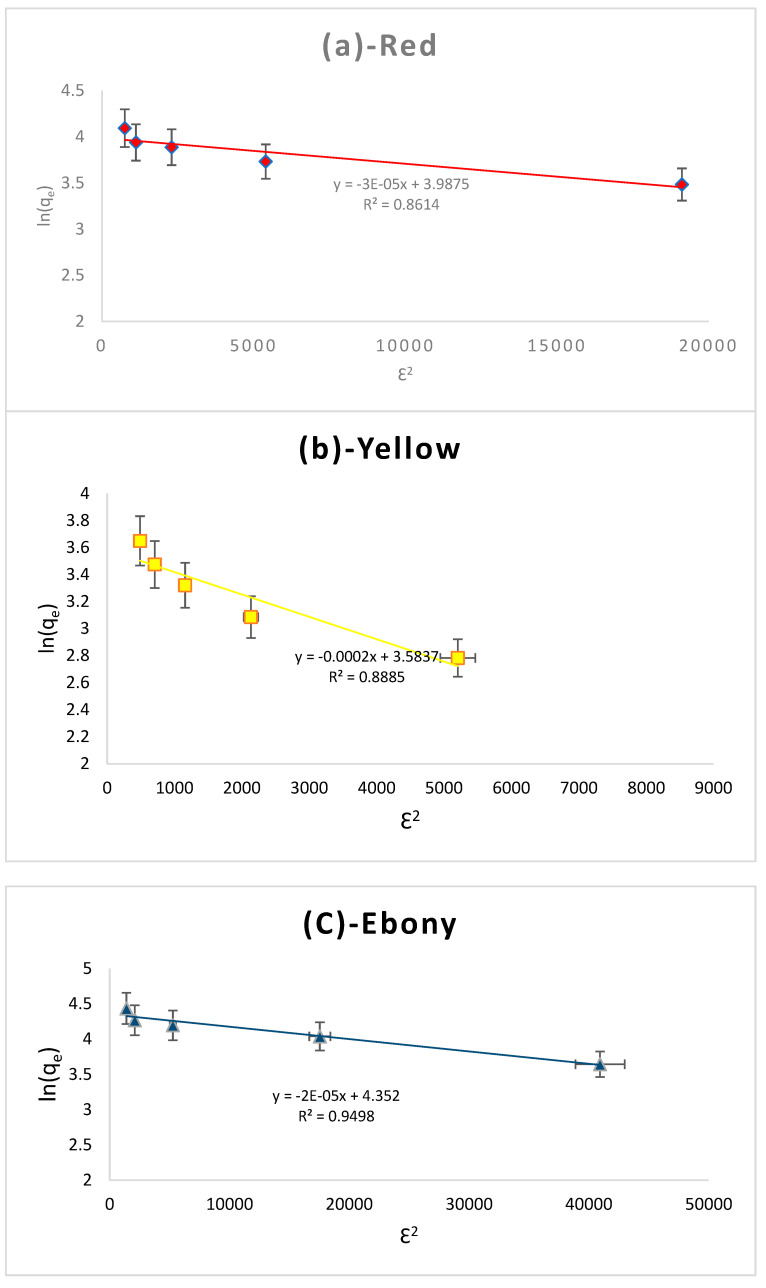
D-R isotherm of (**a**) R, (**b**) Y, and (**c**) E hair dyes onto COZ.

**Figure 17:**
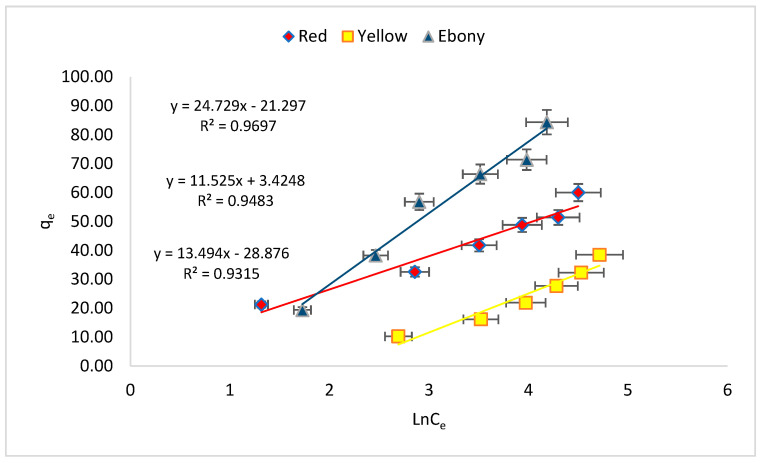
Temkin isotherm of R, Y, and E hair dyes onto COZ.

The D-R and Temkin isotherms are presented in Figure 16 and Figure 17. According to Figure 16, E dye data is more D-R isotherm-adequate than R and Y hair dyes. Following the D-R isotherm, the estimated energy values (less than 8.0 KJ/mol) imply that the adsorption of E dye onto COZ is physisorption in nature [21].

The values of Temkin constant B (Table 3) reveal that the adsorption of hair dyes onto COZ adsorbent is endothermic. The plot demonstrated the endothermic nature of the adsorption process. Interestingly, a robust interaction between the hair dyes (R, Y, and E) and COZ adsorbent is revealed by the linear adequate of the acquired data to the Temkin isotherm [21].

#### 2.2.7. Thermodynamics

The thermodynamic parameters (Gibbs Free Energy Δ*G*°, enthalpy change Δ*H*°_,_ and entropy change Δ*S*°) were computed for the adsorption processes onto COZ. Δ*H*° (kJ mol^−1^) and Δ*S*° (J/mol^−1^ K^−1^) of hair dyes adsorption were computed based on the slope and intercept of the plot of ln (*K_L_*) versus 1/T (K^−1^).

Thermodynamic experiments were performed under the temperature of 25 °C, 35 °C, and 45 °C. The fundamental thermodynamic parameters of the adsorption (Δ*H*°, and Δ*S*°) were calculated from Van’t Hoff’s equation:

(13) $$\ln\left(K_{L}\right)=\frac{∆S^{o}}{R}-\frac{∆H^{o}}{T}\left(\frac{1}{T}\right)$$

The value of Gibbs Free Energy (Δ*G*°) can be obtained by:

(14) $$∆G^{o}=-RT\ln\left(K_{L}\right)$$

where *T* is the temperature in Kelvin (K), *K_L_* is the adsorption equilibrium constant related to the best-fitted model, and *R* is the universal ideal gas constant (8.314 J·K^−1^ mol^−1^).

Negative values of Δ*G*° for the three hair dyes (Table 4) describe the process as favorable and spontaneous. Positive values of Δ*H*° 4.89, 5.06, and 5.92 kJ mol^−1^ for **R**, **Y**, and **E** hair dyes, respectively, confirm the endothermic of the adsorption process. These acquired results are consistent with the Temkin isotherm results. The positive ΔS^o^ values 19.89, 17.01, and 21.25 KJ/mol^−1^K^−1^ predict the randomness at the solid-solution interface during the adsorption process. According to results, the physical and chemical detachments were both concerned with the adsorption of hair dyes onto COZ.

### 2.3. Re-Generation of Adsorbent

**Figure 18:**
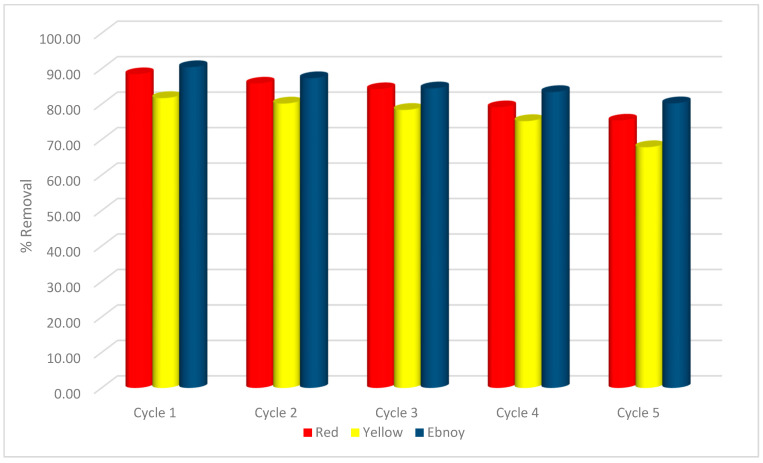
Removal Percentage of R, Y, and E in Regeneration process of COZ adsorbent.

Adsorption/desorption experiments were conducted in an attempt to predict the regeneration of the COZ adsorbent. A 1.0 M of the acetic acid solution was used for R and Y dyes, whereas a 1.0 M NaOH solution was used for the E dye [22]. The adsorption percentage removal after five cycles of re-generation is presented in Figure 18.

The desorption of R and Y is related to the established weak electrostatic attraction between the carboxyl group in acetic acid and cationic dyes, which was beneficial for reducing the interaction between dyes (R and Y) and ZnO. The same justification applies to the reaction of acidic E dye with NaOH base. The pH of the solution is another feature that promotes dye desorption processes. The adsorbent surface turns positive (ZnOH^2+^) when pH < pH_pzc_ and negative (ZnO^−^) when pH > pH_pzc_.

After the five COZ re-generation cycles, the dye uptake declined for R, Y, and E by 13.08%, 13.85%, and 10.20%, respectively. This gradual decrease in removal percentage might well be ascribed to a change in COZ surface physicochemical characteristics. As aforementioned, both physical and chemical adsorption onto COZ were involved. Thus, the chemisorption processes and the very modest dissolution of ZnO at pH = 2 might explain the changes in COZ surface characteristics [23].

**Figure 19:**
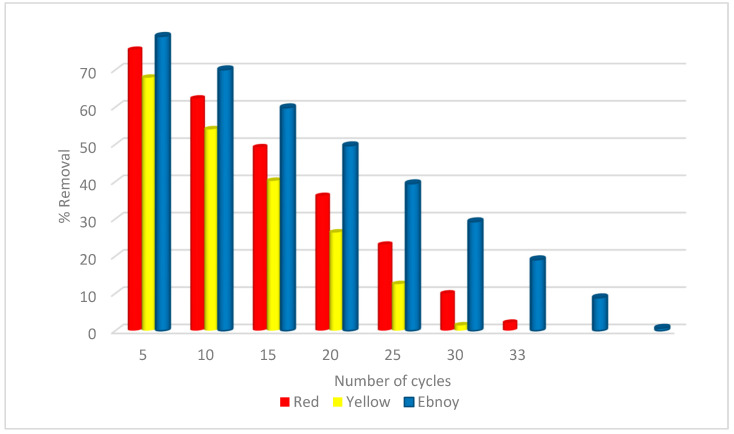
The expected number of cycles to achieve zero capacity of COZ for the three hair dyes.

The number of cycles that lead to zero uptakes of the three hair dyes (R, Y, and E) onto COZ adsorbent (zero capacity of COZ) might reasonably be predicted based on the results in Figure 18. For R, Y, and E hair colors, respectively, it is expected that zero capacity of COZ adsorbent would be achieved after about 33, 29, and 44 cycles (Figure 19). For example, the R dye uptake declined by 13.08% per five cycles, and it was anticipated to decline by 26.16%, 39.24%, 52.32%, 78.48%, 91.56%, 99.408% per 10, 15, 20, 25, 30, and 33 cycles, respectively.

## 3. Material and Methods

### 3.1. Chemicals and Instruments

Three hair dyes; Arianor Madder red 306003 (**R**), Arianor Straw Yellow 306005 (**Y**), Arianor Ebony 306020 (**E**), and ZnSO_4_.7H_2_O were purchased from Sigma Aldrich (Darmstadt, Germany) and used without further purification. Three stock solutions of 1000 mg·L^−1^ concentrations were prepared and then diluted to the required working concentrations (25–150 mg·L^−1^). The pH of solutions was adjusted using 0.1 M NaOH and 0.1 M HCl solutions. The functional groups on the oak cupule powder, O, and oak coated with ZnO, COZ, before and after the adsorption processes were characterized utilizing a TENSOR FTIR instrument from BRUKER. The FT-IR measurements were taken within the wavenumber region of 500–4000 cm^−1^. Adsorbent material was portrayed utilizing scanning electron microscopy (SEM; Model: A Phenom XL G2 scanning electron microscope from Thermo Fisher Scientific, Waltham, MA, USA). With the use of the PANalytical B. V. Lelyweg 17602 EA ALMELO instrument, X-ray diffraction (XRD) investigations were conducted.

### 3.2. Adsorbent Preparation and Characterization

The following methodology was employed to prepare the targeted coated oak cupules (COZ). The oak cupules were collected from the province of Jerash. The cupules were rinsed, dried at 70 °C for about 48 h, powdered, sieved to a size of 125 μm, and stored to be coated with ZnO. A mixture of 5.0 g of oak cupules powder and 60 mL of 0.2 M ZnSO_4_.7H_2_O solution (0.012 moles) was vigorously stirred for about 18 h during the addition of 30 mL 0.8 M NaOH solution (0.024 moles). The coated oak cupules with ZnO (COZ) were filtered, rinsed with distilled water, dried at 100 °C for 2–3 h, and then stored to be used for the removal of hair dyes (R, Y, and E) from aqueous solution. An X-ray diffractometer (XRD, PANalytical B. V. Lelyweg 17602 EA, (Almelo Netherlands) was utilized to get the XRD patterns of the **COZ** structure. The surface morphology images were depicted using the SEM instrument (A Phenom XL G2 scanning electron microscope from Thermo Fisher Scientific). SEM images assisted in showing the surface morphological changes experienced by the formation of adsorbents or by the adsorption process. BRUKER FT-IR spectrometer (Darmstadt, Germany) was utilized to determine the functional groups presence on the oak cupules powder, **COZ**, and their significance in the adsorption process. Thermo Scientific pH-meter was utilized for pH measurements.

### 3.3. Bach Adsorption Experiment

Batch adsorption experiments of the three hair dyes (**R**, **Y**, and **E**) were conducted using **COZ**. The dye solution was delivered in 50 mL increments at various concentrations to a flask with a known mass of adsorbent. The flask contents were shaken for a certain time at a speed of 150 rpm. The solution was then filtered from the adsorbent and subjected to absorbance measurements to estimate concentration. Contact time (30–210 min), initial dye concentration (25–150 mg·L^−1^), adsorbent dose (0.01–0.1 g), and solution pH (3–10) were all investigated. The concentrations of the residual dye in the solutions were ascertained using a UV-6100 Double beam spectrophotometer at wavelengths 500, 402, and 617 nm for R, Y, and E dyes, respectively. The adsorbed amount of the hair dye, q_e_ (mg·g^−1^), was computed using Equation (1).

## 4. Conclusions

Three hair dyes (R, Y, and E) were examined for their capability to adsorb onto COZ adsorbent. The results obtained from the characterization techniques (FT-IR, XRD, and SEM) confirm the coating process of the oak cupules powder by ZnO particles. The following conditions were found to be optimal for the uptake of the three hair dyes: adsorbent mass of 0.07 g, 0.06 g, and 0.05 g for R, Y, and E dyes, respectively, and contact time of 120 min for R and E, and 150 min for Y, with an initial concentration of 50 mg·L^−1^ for the three hair dyes, pH = 9 for R and E, and 7.0 for Y. Adsorption isotherm data for R and Y hair dyes were derived using the Freundlich model, whereas the E hair dye isotherm data fit the Langmuir model. The spontaneity and the endothermic nature of the adsorption process were confirmed by the thermodynamic results. The kinetic data of the **R** and **Y** hair dyes were modeled by the pseudo-second-order, revealing that the nature of the kinetic adsorption is chemical. On the other hand, the kinetic data of the **E** hair dye was modeled by the pseudo-first-order. The rapid adsorption kinetics and high uptake values indicated that COZ is a highly competitive adsorbent for the removal of R, Y, and E hair dyes from solutions. Experiments proved that COZ adsorbent could be re-generated using acetic acid for R and Y recovery, and NaOH for E.

## Figures and Tables

**Table 1:** FTIR Results.

	Wavenumber (cm^−1^)				
	O	COZ	COZR	COZY	COZE
O–H stretching vibration	3332.57	3354.74	3307.87	3560.62	3332.60
aliphatic C–H group stretching vibrations of the −CH_3_ and −CH_2_ groups	2925.52	2944.77	2967.64	2923.73	2878.23
stretching vibration of C=O of carboxylic groups	1738.09	1740.43	1594.97	1730.56	1723.53
carboxylic/aromatic hydroxyl (−OH) stretching of phenol group	1435.4 and 1221.38	1418.70 1116.59	1417.06 1260.86	1363.93 1113.43	1458.67 1108.81
C=C, the C–H bond, and O–H in the plane deformation	902.42	1064.24	1024.78	1020.21	1029.60
C–H deformation vibration and CH_2_ rocking vibration −C–N– and −C–C– stretching	(525.89–902.42)	(516.48–1064.24)	(600.88–1024.78)	(600.78–1020.21)	(672.06–1029.60)
Bond to Metal oxide (ZnO)	459.25	410.13	412.86	450.60	519.92

**Table 2:** Kinetics constants.

	First Order Kinetics		Second Order Kinetics	
	R^2^	K_1_ (min^−1^)	R^2^	K_1_ (g·mg^−1^min^−1^)
R hair dye	0.7344	−5.508 × 10^−3^	0.9992	1.88 × 10^−2^
Y hair dye	0.4312	−8.40 × 10^−3^	0.9373	−6.7 × 10^−3^
E hair dye	0.9964	+1.7764	0.4077	0.0269

**Table 3:** Isotherms model constants.

	Langmuir				Freundlich			D-R			Temkin		
Hair Dye	$R_{L}^{2}$	R_L_	K_L_	q_m_ _mg/g_	$R_{F}^{2}$	K_F_ _mg/g_	n	R^2^	B_D_ mol^2^/KJ^2^	E KJ/mol	R^2^	B	A
Red	0.9239	0.1823	0.3310	55.2	0.9905	13.72	3.15	0.8614	3 × 10^−5^	0.13	0.9697	11.5	1.3
Yellow	0.9748	0.5250	0.01673	52.6	0.9942	1.70	1.53	0.8885	2 × 10^−4^	0.05	0.9315	13.5	0.12
Ebony	0.9827	0.3989	0.03345	135.1	0.9494	11.97	2.57	0.9498	2 × 10^−5^	0.16	0.9483	24.7	0.42

**Table 4:** Thermodynamic parameters of adsorption of hair dyes (R, Y, and E) onto COZ.

Hair Dye	T (K)	q_m_ (mg·g^−1^)	Thermodynamic Parameters		
			∆G° (KJ mol^−1^)	∆H° (KJ mol^−1^)	∆S° (KJ K^−1^mol^−1^)
Red	298	35.10	−3.61	4.89	19.89
Yellow	308	43.39	−3.78		
Ebony	318	52.47	−4.65		
Red	298	24.00	−0.24	5.06	17.01
Yellow	308	32.21	−0.73		
Ebony	318	50.60	−1.31		
Red	298	39.66	−1.32	5.92	21.25
Yellow	308	42.43	−2.17		
Ebony	318	51.68	−2.57		

## Data Availability

The data are available within the body of the article.
